# Increased Pathogenicity of West Nile Virus (WNV) by Glycosylation of Envelope Protein and Seroprevalence of WNV in Wild Birds in Far Eastern Russia

**DOI:** 10.3390/ijerph10127144

**Published:** 2013-12-12

**Authors:** Hiroaki Kariwa, Ryo Murata, Masashi Totani, Kentaro Yoshii, Ikuo Takashima

**Affiliations:** 1 Laboratory of Public Health, Department of Environmental Veterinary Sciences, Graduate School of Veterinary Medicine, Hokkaido University, Kita-18, Nishi-9, Kita-Ku, Sapporo 060-0818, Japan; E-Mail: kyoshii@vetmed.hokudai.ac.jp; 2 Laboratory of Animal Health, Department of Animal Science, Faculty of Agriculture, Tokyo University of Agriculture, Funako 1737, Atsugi 243-0034, Japan; E-Mail: r3murata@nodai.ac.jp; 3 Third Animal Quarantine Division, Animal Quarantine Service, Narita Branch, Ministry of Agriculture, Forestry and Fisheries, Ohaza Tennami, Sanrizuka, Aza Nishihara 254-1, Narita 282-0011, Japan; E-Mail: smallplums@frontier.hokudai.ac.jp; 4 Department of Nutrition, Faculty of Nursing and Nutrition, Tenshi College, Kita-13, Higashi-3-1-30, Higashi-Ku, Sapporo 065-0013, Japan; E-Mail: takasima@vetmed.hokudai.ac.jp

**Keywords:** West Nile virus, Japanese encephalitis virus, flavivirus, envelope protein, glycosylation, pathogenicity, replication, chick, neutralizing antibody, seroprevalence

## Abstract

In this review, we discuss the possibility that the glycosylation of West Nile (WN) virus E-protein may be associated with enhanced pathogenicity and higher replication of WN virus. The results indicate that E-protein glycosylation allows the virus to multiply in a heat-stable manner and therefore, has a critical role in enhanced viremic levels and virulence of WN virus in young-chick infection model. The effect of the glycosylation of the E protein on the pathogenicity of WN virus in young chicks was further investigated. The results indicate that glycosylation of the WN virus E protein is important for viral multiplication in peripheral organs and that it is associated with the strong pathogenicity of WN virus in birds. The micro-focus reduction neutralization test (FRNT) in which a large number of serum samples can be handled at once with a small volume (15 μL) of serum was useful for differential diagnosis between Japanese encephalitis and WN virus infections in infected chicks. Serological investigation was performed among wild birds in the Far Eastern region of Russia using the FRNT. Antibodies specific to WN virus were detected in 21 samples of resident and migratory birds out of 145 wild bird samples in the region.

## 1. Introduction

The West Nile (WN) virus is a mosquito-borne flavivirus of the Japanese encephalitis (JE) serocomplex group that causes lethal encephalitis in humans and horses. WN virus was first isolated in 1937 from the blood of a febrile patient in the WN district of Uganda [1]. WN virus has since been found to be endemic over a wide range of areas in Africa, the Middle East, Western Asia, and Australia [2,3,4]. Outbreaks of various magnitudes occurred in Israel in 1941 and 1951–1954 and in Africa in 1974. After that, no large outbreaks were observed for 20 years; however, from 1994 to 2000, WN outbreaks occurred among humans and horses [5]. Specifically, outbreaks occurred in Algeria in 1994, in Morocco in 1996, in Romania in 1996, in Tunisia in 1997, in the Czech republic in 1997, in the Congo in 1998, in Italy in 1998, in Israel from 1997 to 2000, in Russia in 1999, in France in 2000, and in the United States from 1999 to the present [6]. In the early outbreaks of the 1990s, the WN virus was associated only with mild pathogenicity to avian and mammalian hosts. However, during the latter half of the 1990s, new strains of WN virus emerged in Europe. Humans and horses infected with those strains frequently suffered from meningitis and encephalitis [5]. Since the outbreak of WN encephalitis in humans and horses in New York City (NYC) in late August 1999, the WN virus has spread throughout North America and has very rapidly expanded to South American countries. Endemic areas are still expanding.

The WN virus endemic in North America was characterized by large-scale mortality in wild birds [7], particularly in corvids, a phenomenon that had not been observed before the outbreaks in New York City (NYC) and Israel [5]. A single nucleotide change resulting in the T249P substitution in the NS3 helicase was reported to be associated with large-scale mortality in American crows [8]. WN virus is maintained in nature through an enzootic transmission cycle between avian reservoir hosts and *Culex* mosquito vectors. Viremic levels of the avian host directly affect the infection rates of vector mosquitoes; birds with higher viremia generate more infected mosquitoes after blood feeding [9]. Replication and dissemination characteristics of the virus within the mosquito vectors also affect transmission efficiency.

The flavivirus envelope (E) protein is an important structural protein in virus–cell interactions, and it is a major target of the host-antibody responses [10]. All flaviviruses have one or two potential N-linked glycosylation sites on the E protein [11]. Some WN viruses contain the N-linked glycosylation motif (N-Y-T/S) at residues 154–156 of the E protein, whereas others lack this glycosylation site because of amino acid substitutions. It is interesting to note that many of the WN virus isolates associated with significant human outbreaks, including the recent North American epidemic, possess the glycosylation site on the E protein [12]. In a previous study, we isolated four variants from two WN virus NYC strains using plaque purification on baby-hamster kidney (BHK) cells [12]. Two of the variants contained glycosylated E proteins, whereas the others contained non-glycosylated E proteins. To determine the relationship between E-protein glycosylation and pathogenicity of the WN virus, mice were inoculated subcutaneously with these four variants. The glycosylated variants caused higher mortality than the nonglycosylated variants in mice, which suggests that E-protein glycosylation is a molecular determinant of neuroinvasiveness in the NY strains of WN virus. Other studies also established the importance of glycosylation of flaviviruses E protein for viral assembly and infectivity *in vitro* and *in vivo* [12,13,14].

When an outbreak of WN virus occurred in and around NYC in 1999, many wild and exotic birds died, and encephalitis in humans and horses was reported [15,16]. Recently, highly pathogenic WN virus has been reported in Africa, America, Europe, and Russia, and it has become a public health concern [17]. Birds play an important role in the transmission of WN virus; thus, knowledge of the pathogenicity of WN virus in birds is vital for the control and prevention of infections with this virus. Susceptibility to WN virus varies by bird species. During the 1999 NYC outbreak, various species of birds died, including crows, flamingos, and eagles [18,19,20,21]. Most deaths in wild birds have been in the order Passeriformes (crows and jays). American crows (*Corvus brachyrhynchos*) [22] and young domestic geese (*Anser anser domesticus*) [23] subsequently showed high susceptibility and mortality with a high level of viremia when infected with NYC isolates. WN virus infection in wild birds causes depression, weight loss, and occasionally neurologic signs such as ataxia, tremors, and torticollis in highly susceptible species [21,23]. WN virus has been isolated from multiple organs, such as the brain, heart, spleen, liver, and kidney, and encephalitis and myocarditis have been reported [21,23,24,25].

Sequence analysis of various WN virus strains has shown that recent highly pathogenic WN virus isolates, such as the NYC isolate, have a glycosylation site in the E protein. Since young domestic chick is susceptible to WN virus [26], it is important to evaluate the pathogenicity of glycosylated and nonglycosylated WN virus variants in chicks in detail.

After the outbreak of WN encephalitis in humans and horses in NYC in 1999, WN virus has spread throughout North America very rapidly [5]. In European Russia, WN virus was first isolated from humans and ticks in 1963. In 1999, 318 confirmed cases of human infection with WN virus were reported in the Volgograd Region, resulting in 40 deaths [27,28]. In 2004, WN virus was reported in patients in Novosibirsk in the southwest region of Siberia [29]. West Nile virus has shown a tendency to spread eastward through Russia. It is possible that migratory birds have carried the virus from Far East Russia to East Asian countries during migration. The JE virus is endemic to East Asia. Although pigs are the amplifier hosts of the JE virus, wild birds may serve as the reservoir host. JE and WN viruses are closely related and often display serological cross-reactivity [30,31]. The geographical distributions of JE and WN viruses rarely overlap; however, as WN virus continues to spread, both viruses may infect wild birds, which are a common host. Therefore, a diagnostic test that can distinguish between WN and JE virus infections is required. Plaque reduction neutralization test (PRNT) is the golden standard for the specific antibody detection in flavivirus infections. However, PRNT requires certain amount of serum and is not suitable for serological survey. The micro focus reduction neutralization test (FRNT) method has several advantages over the PRNT; a large number of serum samples can be handled at once and the test can be performed on a small volume (15 μL) of serum. To investigate cross-reactivity to heterologous virus infection in micro FRNT, an infection experiment was performed by inoculation of WN or JE virus to chicks. The seroprevalence of WN virus among wild birds in the Far Eastern region of Russia was investigated using the FRNT.

## 2. Increased Pathogenicity of West Nile Virus by Glycosylation of Envelope Protein in Chicks

**Figure 1:**
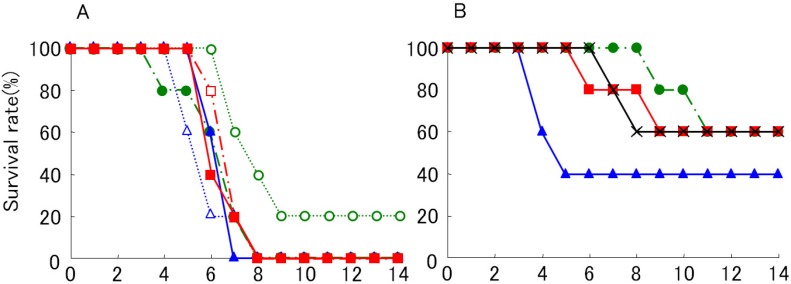
Survival curves of young chicks subcutaneously inoculated with WN virus 6-LP (**A**) and 6-SP (**B**) variants. Two days old male chicks were inoculated with 10^−1^ (○), 10^0^ (△), 10^1^ (☐), 10^2^ (●), 10^3^ (▲) and 10^4^ (■) PFU of 6-LP, and 10^2^ (●), 10^3^ (▲), 10^4^ (■) and 10^5^ (×) PFU of 6-SP. Chicks were observed daily for health conditions. The number of chicks used was 5 for each variants.

Previously, we plaque-purified four WN virus variants that had different amino acid sequences at the N-linked glycosylation site in the E-protein sequence [12]. The E protein was glycosylated in two of these strain variants. The glycosylated variants produce large plaques (LP), and the nonglycosylated variants produce small plaques (SP) in BHK cells. The LP variants are more pathogenic in mice than are the SP variants [12]. Most of the strains occurring before the 1990s and some of the low pathogenic strains do not have the N-glycosylation site, whereas many of the highly pathogenic strains that emerged recently have the N-glycosylation site [12]. A rare WN virus isolated in Mexico lacks the glycosylation site on the E protein, and it was shown to have reduced pathogenicity in mice [32]. N-linked glycosylation of the WN virus E protein was previously shown to be responsible for enhanced neuroinvasiveness of the virus in a mouse model [12,14,33]. However, few studies [34] have been conducted to determine the role of glycosylation of the E protein in WN virus dynamics in birds and mosquitoes, both natural hosts of the virus. We found that young chicks can serve as a model to study the pathogenicity of WN virus in avian hosts [26]. Subcutaneously injected LP variants resulted in a much higher mortality rate (LD_50_ < 0.1 PFU) than SP variants (Figure 1), suggesting that glycosylation of the E protein of WN virus is a determinant of pathogenicity in chicks that have been peripherally inoculated. 

**Figure 2:**
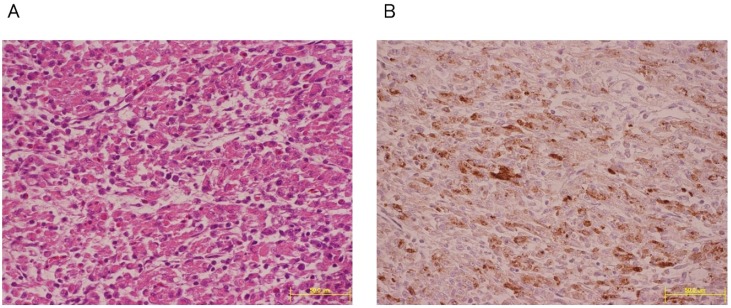
Histopathological and immunohistochemical findings of the 6-LP infected young chicks. (**A**) Photomicrograph of marked necrosis of myocytes of heart from a young chick with WN virus infection. HE stain. (**B**) Myocytes of heart are positively stained for WN virus antigen.

**Figure 3:**
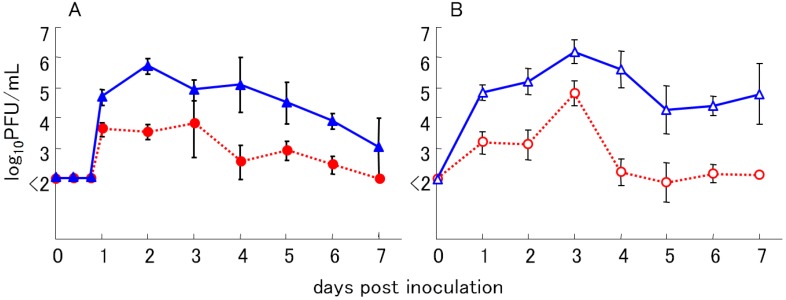
Viremic levels of young chicks subcutaneously inoculated with WN virus variants. Young chicks were inoculated with WN virus variants, 6-LP (▲) and 6-SP (●) in experiment (**A**), and B-LP (△)and B-SP (○) in experiment (**B**). Two days old chicks were inoculated with 100 PFU of all variants (n = 4). The virus titers in sera were measured by plaque assay on BHK cells. Mean (±SD) titers are from triplicate cultures.

Histopathological findings in dead chicks included necrosis in hepatocytes and necrotic myocarditis, and cardiovascular failure was the suspected cause of death in these birds (Figure 2). These histopathological changes were also seen in birds that had been naturally infected with WN virus [35]. Efficient viral propagation both in avian and mosquito hosts is an important determinant of active viral circulation in the natural transmission cycle. The viremic levels of chicks inoculated with LP variants were higher than those inoculated with SP variants (Figure 3). The viremic titers of chicks inoculated with LP variants exceeded 10^5^ plaque forming unit (PFU)/mL blood during 2–4 days post inoculation. Previous studies showed that avian viremic levels higher than 10^5^ PFU/mL are crucial for the efficient infection of vector *Culex tritaeniorhynchus* mosquitoes [9]. These results showed that N-linked glycosylation of WN virus E protein is a determinant of high viremic levels in young chicks and suggest that glycosylated WN-virus variants may be more efficiently transmitted to vector mosquitoes than non-glycosylated variants because of higher viremia in infected birds.

### 2.1. Increased Replication of Glycosylated WN Virus Variant in Vitro

**Figure 4:**
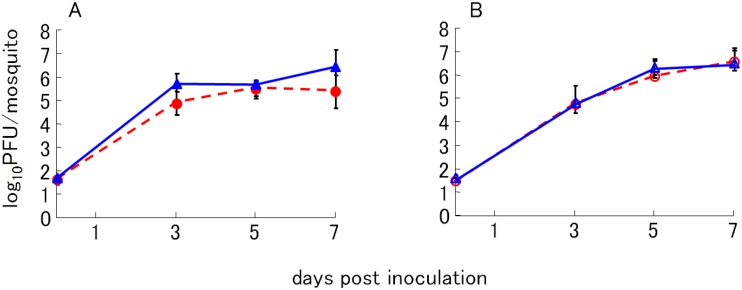
Virus titer of WN virus variants in *Culex pipiens pallens.* Seven days old female mosquitoes (n = 4) were inoculated intrathoracically with 100 PFU of all variants. The virus titers in mosquito bodies were measured by plaque assay on BHK cells. The virus titers of 6-LP (▲) and 6-SP (●) were shown in (**A**) and those of B-LP (△) and B-SP (○) were shown in (**B**).

To explain the differences in viremic titers of chicks inoculated with the two variants, growth characteristics of the LP variant, which is glycosylated, and the SP variant, which is not glycosylated, were examined in tissue culture cells at different temperatures. The results suggest that glycosylation of the E protein imparted heat stability to WN virus during propagation in cells at high temperature (data not shown). We tested three kinds of cultured cells, namely BHK cells from a mammalian host, QT6 cells from an avian host, and C6/36 cells from mosquitoes, each representing an important host in the natural transmission cycle of WN virus. Viral growth characteristics were examined by culturing the cells at different temperatures. Compared with LP variants, SP variants showed a remarkable reduction in viral growth in BHK cells at 37 °C and 40 °C and in QT6 cells at 40 °C and 42 °C [26]. Reduction rates of viral titers in the culture media without cells were not significantly different between SP and LP variants. Collectively, differences in the heat-stable characteristics of the LP variants and the heat-labile characteristics of the SP variants in BHK cells and QT-6 cells at high temperatures depended on the glycosylation status of the E protein of the variants, which affected the viral-replication steps within the cells. In contrast, no significant differences in viral titers were observed between the LP and SP variants when *Culex pipiens* mosquitoes were inoculated intrathoracically with each variant (Figure 4) [26]. The disseminated infection rates of mosquitoes orally infected with the variants did not show any difference between the LP and SP variants (Table 1). Moreover, there were no differences in the propagation of the two variants in C6/36 cells at various temperatures. The results suggest that the glycosylation status of the E protein may not affect viral propagation and dissemination in mosquitoes. The similar in vitro replication properties were also observed in WN virus NYC strains with glycosylated and nonglycosylated E protein generated by reverse genetic technology [36].

**Table 1:** Disseminated infection rates of *Culex pipiens* during peroral infection experiments with WN virus.

WN virus variant	Virus dose (PFU)		
	10^7^	10^6^	10^5^
6-LP	10/10 *	9/9	5/6
6-SP	6/6	11/11	4/10

Note: * Number of virus positive mosquitoes; / Number of inoculated mosquitoes, Mosquitoes were fed upon blood-virus mixture and kept at 28°C for 13 days, harvested and titrated for virus on BHK cell plaque assay.

We previously examined the role of the N-linked glycans of E protein in tick-borne encephalitis (TBE) virus particle secretion using subviral particles [13]. Secretion of virus particles was greatly reduced in culture cells transfected with mutant vectors that have an amino acid substitution of T156A in the E protein, and the study also suggested that the reduced particle secretion is caused by glycan loss rather than to the amino acid substitution per se. The amino acid substitution of T156A in TBE virus is similar to that of S156P in WN virus in terms of amino acid characteristics, and both mutations altered the protein such that it would not be recognized by oligosaccharyl-transferase [37]. Collectively, the observed differences between LP and SP variants are most likely caused by glycan loss on the E protein rather than to the amino acid substitutions. Our previous study [13] using a subviral system of tick-borne encephalitis virus showed that a mutant lacking E-protein glycosylation has a large reduction in the level of secretion of the E protein; the E protein is retained at the endoplasmic reticulum and is rarely present in the Golgi complex. In the dengue virus, this glycosylation at aa154 occurs in E-protein domain I, close to the center of the fusion peptide of E-protein domain II, and glycosylation of the E protein is considered to increase the stability of the protein [38,39]. Glycosylation of the E protein of WN virus may also be important for the folding and stability of the viral protein at high temperatures.

Mutations of NS3 or NS4B of the NY strain of WN virus were reported to be responsible for the increased pathogenicity and viremic level in avian or mammalian hosts [8,40]. Importantly, the introduction of a T249P amino acid in NS3 helicase was shown to be crucial for the above-mentioned viral characteristics. We showed that N-glycosylation of the E protein facilitated efficient multiplication of the NY strain of WN virus at high temperatures in an avian cell culture, and it was responsible for the higher viremic level in an avian host. The observation that most recent isolates of lineage I WN virus carry the N-glycosylation site on the E protein [12] suggests that glycosylation of the E protein is a pre-requisite for the stable circulation of WN virus in the avian–mosquito transmission cycle, and it may be one of the multiple determinants for efficient transmission. However, there may be other factors for efficient WN virus transmission in nature. In the 1980s, Kunjin (KUN) viruses, which are an Australian variant of WN virus, were not glycosylated in E protein [41], while in the 1990s glycosylated KUN virus isolates were found [42,43]. Russian WN virus isolated in 1999 at Volgograd was also nonglycosylated [28]. The circulating virus strains may be influenced by environmental factors such as vectors and host species as well as the existence of other WN virus strains.

### 2.2. Replication of WN Virus and Cytokine Responses in Infected Chicks

**Figure 5:**
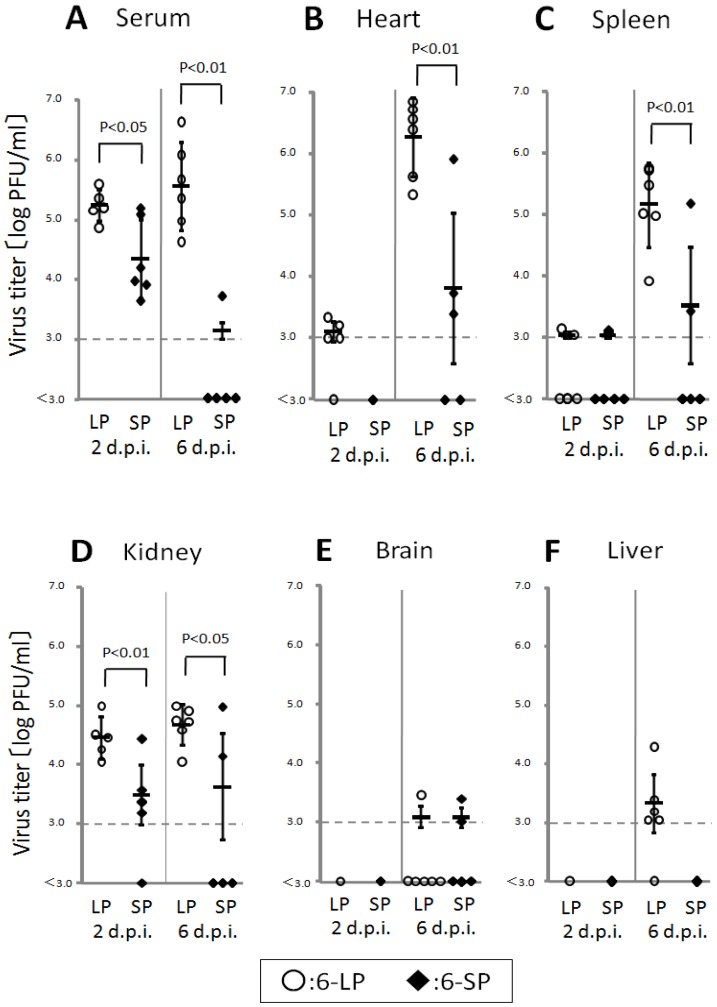
Viral titers in the serum (**A**), hearts (**B**), spleens (**C**), kidneys (**D**), brains (**E**), and livers (**F**) of 2-day-old chicks infected with 6-LP (○) or 6-SP (◆). Chicks were infected with 10^2^ PFU subcutaneously (s.c.) in the femoral region, and blood and tissues were collected at 2 and 6 days post infection (d.p.i.). Virus titers were determined by plaque assay using BHK-21 cells (n = 5 or 6). Individual and mean PFU values are represented by symbols and bars, respectively. When mean values were calculated, the titers of samples below the detection limit (10^3^ PFU/mL) were considered to be 3.0. Error bars indicate standard deviations. *p*-values were calculated using unpaired Student t-tests.

Birds play an important role in the transmission of WN virus in nature; however, the pathogenicity of this virus in birds remains unclear. Thus, understanding the transmission and pathogenicity of WN virus in birds is vital for the establishment of efficient preventive measures. Young domestic chicks were infected with WN virus, and the effect of E protein glycosylation on pathogenicity was determined. The glycosylated variant caused high viremia (>10^5^ PFU/mL) in 2-day-old chicks, and high levels of virus were detected in the hearts, spleens, and kidneys (Figure 5) [44]. In contrast, lower viremia and low levels of virus in organs were observed in chicks infected with the nonglycosylated variant (Figure 5). These data indicated that the glycosylation of the E protein is important for multiplication in peripheral organs. High levels of viremia were also reported in American crows [9,22]. Previous studies [45] have shown that avian viremic levels exceeding 10^5^ PFU/mL are crucial for efficient infection of the insect vector, *Culex pipiens* mosquitoes. Therefore, these data indicate that young domestic chicks may contribute to the transmission of WN virus in nature.

The highest virus titers were detected in the heart of chicks infected with 6-LP (Figure 5), and severe necrotic myocarditis in the hearts of 2-day-old chicks infected with 6-LP were observed [26]. WN virus multiplication and various degrees of cardiac lesions have been reported in dead wild birds [21,25]. These data indicate that the heart is one of the major targets of WN virus in birds. Virus was also detected in the spleens and kidneys of dead wild birds and in young chicks experimentally infected with WN virus. Thus, viral multiplication in peripheral organs, particularly the heart, contributes to the pathogenicity of WN virus in birds.

No virus was detected in the brains, and no neurologic signs were observed in 2-day-old chicks infected with WN virus. Encephalitis has been reported in WN virus-infected mammals [46,47] and several species of birds (e.g., American crows [22] and young domestic geese [23]. These differences indicate that the neuroinvasiveness of WN virus varies depending on the species.

Higher levels of virus were detected in the blood and peripheral organs of 2-day-old chicks infected with the glycosylated WN virus variant. Glycosylation of the E protein has been reported to enhance viral multiplication in mammalian and avian cells [12,14,26,34] and to be involved in the stability of the virion at mildly acidic pH [14]. In a mouse model, glycosylated WN virus caused stronger viremia and higher neuroinvasiveness than did nonglycosylated WNV, resulting in the enhanced virulence [12,14]. Glycosylation of the E proteins was shown to increase mortality in young domestic chicks [26]. In addition, Mexican WN virus strain lacking the glycosylation site in E protein exhibited lower level of viremia and an attenuated phenotype [48]. The Mexican WN virus mutated by reverse genetics having the glycosylation site in E protein together with a mutation in pre-membrane gene showed higher viremic level and higher pathogenicity in wild birds. Therefore, glycosylation of the E protein of WN virus enhanced viral multiplication in peripheral organs, leading to the strong pathogenicity of the virus in birds.

**Figure 6:**
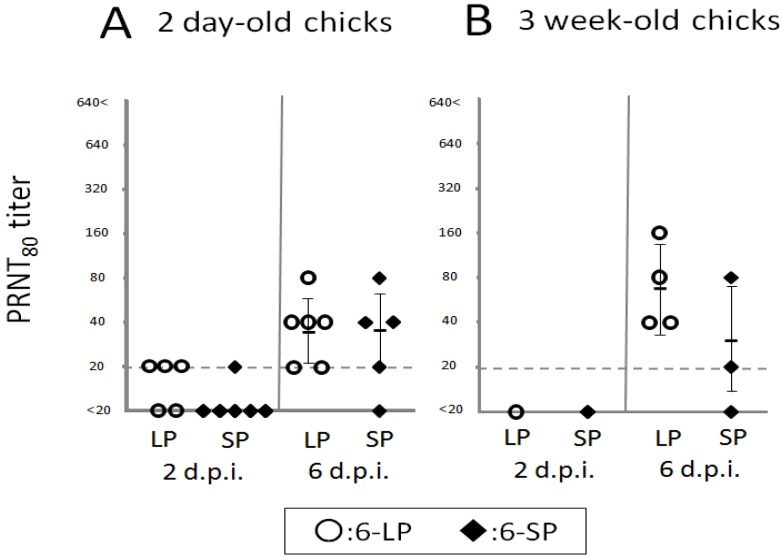
Primary neutralizing antibody responses in chicks inoculated with WNV 6-LP or 6-SP. Two-day-old (**A**) and 3-week-old (**B**) chicks (n = 3–6) were inoculated with 10^2^ PFU of WNV 6-LP (○) or 6-SP (◆). WNV neutralizing antibody titers were then measured by PRNT_80_. Individual and mean PRNT_80_ titers are represented by symbols and bars, respectively. When mean values were calculated, the titers of samples under the detection limit were considered to be 20.

**Figure 7:**
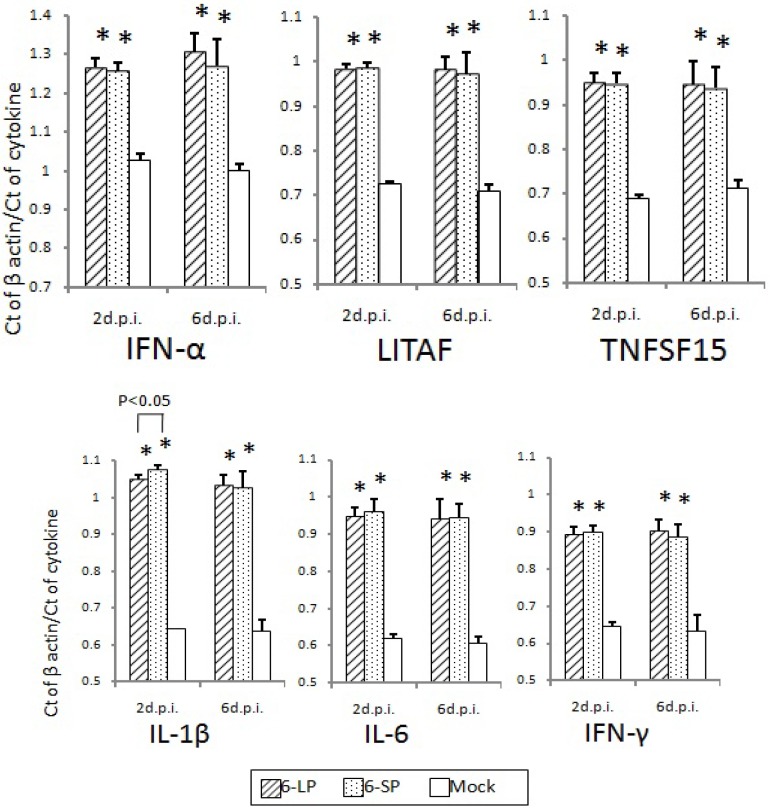
Cytokine and transcription factor mRNA levels in the hearts of 2-day-old chicks inoculated with WNV 6-LP or 6-SP. Chicks were infected with 10^2^ PFU of virus administered subcutaneously in the femoral region, and tissues were collected at 2 and 6 d.p.i. Total RNA was then extracted and cDNA synthesized. SYBR Green-based quantitative real-time PCR was performed using the synthesized cDNA. Relative quantification of cytokine gene expression was done using the CT method. The CT data for each cytokine were normalized against the b-actin levels in the same sample. * and ** indicate statistically significant differences (* *p*, 0.01; ** *p*, 0.05) in cytokine and transcription factor mRNA levels compared with mock-infected chicks.

**Figure 8:**
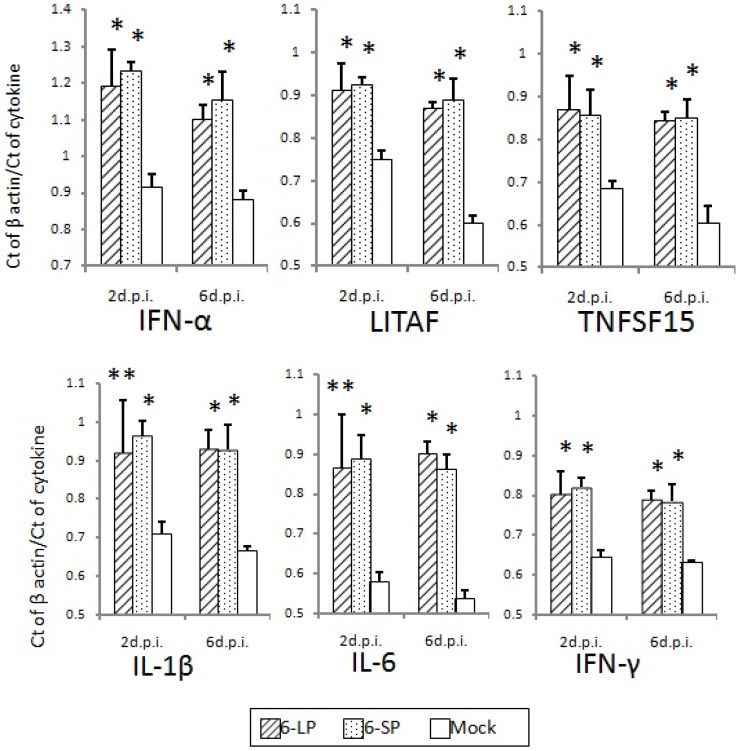
Cytokine and transcription factor mRNA levels in the spleens of 2-day-old chicks inoculated with WNV 6-LP or 6-SP. Chicks were infected with 10^2^ PFU of virus administered subcutaneously in the femoral region, and tissues were collected at 2 and 6 days post infection (d.p.i.).Total RNA was then extracted and cDNA synthesized. SYBR Green-based quantitative real-time PCR was performed using the synthesized cDNA. Relative quantification of cytokine gene expression was done using the CT method. The CT data for each cytokine were normalized against the b-actin levels in the same sample. * and ** indicate statistically significant differences (* *p*, 0.01; ** *p*, 0.05) in cytokine and transcription factor mRNA levels compared with mock-infected chicks.

**Figure 9:**
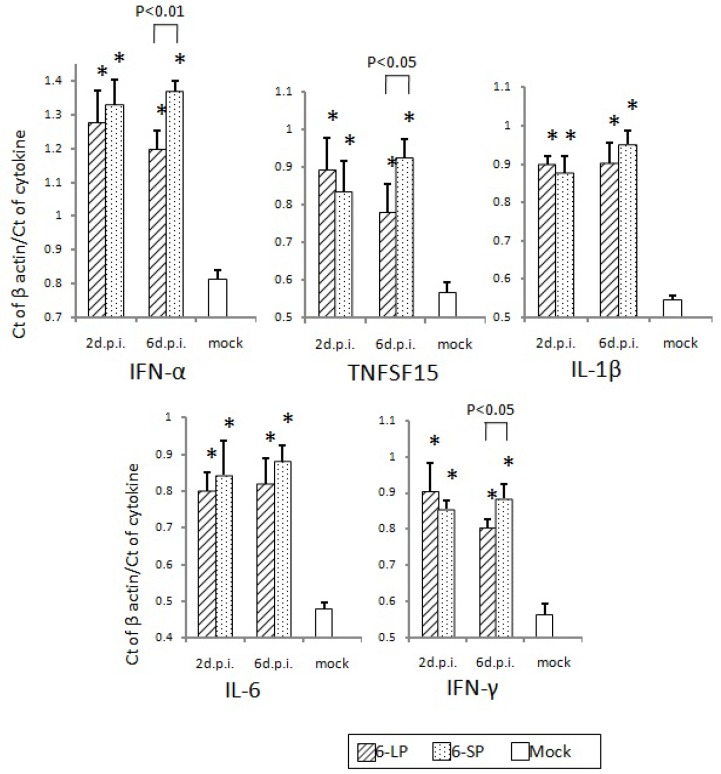
Cytokine and transcription factor mRNA levels in the spleens of 3-week-old chicks inoculated with WNV 6-LP or 6-SP. Chicks were infected with 10^2^ PFU subcutaneously (s.c.) in the femoral region, and tissues were collected at 2 and 6 days post infection (d.p.i.). Total RNA was extracted and cDNA synthesized. SYBR Green-based quantitative real-time PCR was performed using the synthesized cDNA. Relative quantification of cytokine gene expression was done using the CT method. The CT data for each cytokine were normalized against the b-actin levels in the same sample. * Statistically significant differences (*p* < 0.01) in cytokine and transcription factor mRNA levels compared with mock-infected chicks.

Host immune responses were not significantly different in 2-day-old and 3-week-old chicks after infection with either 6-LP or 6-SP. No difference was observed in induction of neutralizing antibodies in chicks infected with glycosylated and nonglycosylated WN viruses (Figure 6). In addition, mRNA levels of cytokines and transcription factors such as IFN-α, LITAF, TNFSF15, IL-1β, IL-6, and IFN-γ were equivalent between chicks infected with glycosylated and nonglycosylated viruses (Figure 7, Figure 8 and Figure 9). In several mouse model studies, the involvement of various proinflammatory cytokines in pathogenicity has been reported, such as immune-mediated tissue damage caused by the expression of TNF-α [49] and the protection against WN virus by TNF-α [50], IFN-γ [51], and IFN-α/β [52]. However, our data indicate that the immune response may not affect the pathogenicity of, or protection against, WN virus infection in birds. Since the cytokine response to viral infection in birds is not well understood, it is possible that other cytokines or chemokines are involved in the response to WN virus infection.

No virus was detected in the blood and organs of 3-week-old chicks, although neutralizing antibodies and cytokine responses were induced. These data indicate that the virus was cleared at an early stage of infection, prior to multiplication in organs. A similar low susceptibility to WN virus was reported in older chicks and adult chickens [17,53,54]. It is thus possible that susceptibility to WN virus changed as the chicks grew, leading to lower viral multiplication.

In summary, the glycosylated variant of WN virus was highly pathogenic in 2 day-old chick, indicating the utility of the young chick model of WN virus infection. Glycosylation of the E protein was shown to enhance viral multiplication in the blood and peripheral organs, which is itself associated with high pathogenicity. These findings will contribute to a greater understanding of WNV pathogenicity in birds and will facilitate more effective control measures and the prevention of WN virus infection.

### 2.3. Establishment of Micro-Focus Reduction Neutralization Test to Detect Antibodies to WN Virus

In recent years, the geographic distribution of WN virus has expanded rapidly to various parts of the world [5]. When WN virus spreads to a non-endemic area, a differential diagnosis with a closely related flavivirus is required. The JE virus, which belongs to the same serocomplex as WN virus, is distributed throughout East Asian countries, and the viruses are serologically cross-reactive [30,31]. Therefore, micro focus reduction neutralization test (FRNT) was evaluated for effective differential sero-diagnosis of JE and WN virus infection in birds.

Young chicks were used for the WN virus infection experiment, as a model of wild birds [9,17,53,54]. Although wild birds are natural hosts of JE virus, similar to WN virus, few instances of JE virus infection in birds have been reported [55,56]. Two-day-old chicks were inoculated with WN or JE virus and blood were collected. Viremia was measurable in all inoculated chicks with the maximum viremia titer reached 10^4^ PFU/mL. These results suggest that the young chicks infected with JE virus or WN virus were an effective animal model for infection by both viruses.

**Figure 10:**
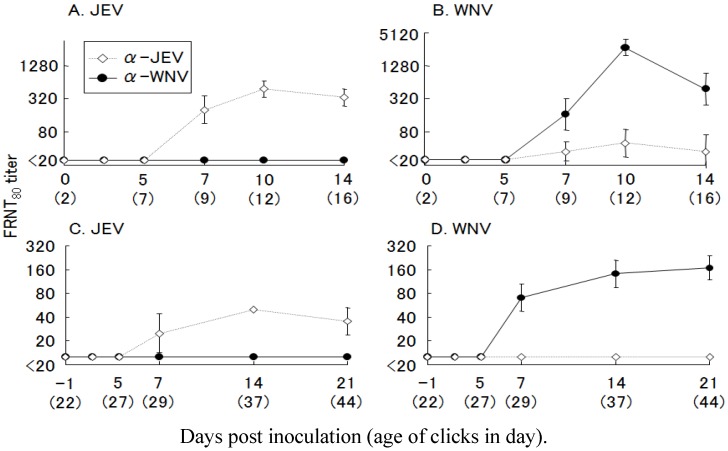
Primary neutralizing antibody responses in chicks inoculated with Japanese encephalitis (JE) and West Nile (WN) viruses. Two-day-old chicks (*n =* 4) were inoculated with 100 plaque forming units (PFU) of (**A**) JE virus and (**B**) WN virus, and 3-week-old chicks (*n =* 4) were inoculated with 1,000 PFU of (**C**) JE virus and (**D**) WN virus. Anti-JE virus (◇) and -WN virus (●) neutralizing antibody titers were measured by FRNT_80_ and are expressed as the mean ± SD.

Next, we inoculated 2-day-old and 3-week-old chicks with JE or WN virus and measured the antibody response. After single-virus infection, only neutralizing antibodies specific to the homologous virus were detected in the chicks (Figure 10). In 3-week-old chicks, the antibody responses were low compared with those of the 2-day-old chicks. Adult galliformes have a low susceptibility to WN virus, and viremia titers in these birds have been reported to be lower than those of young birds [57,58]. Because the 3-week-old chicks were older, the immunological response to JE virus infection in these birds was weaker than in 2-day-old chicks, but in this study, antibody titers sufficient for evaluation of the FRNT were obtained. Most sera from the infected chicks showed 4-hold or greater FRNT titers to the homologous viruses. We, therefore, adopted 4-hold or greater difference in FRNT titer as the standard for the specific antibody to either to WN or JE virus. To study the effect of heterologous virus infection, a double-infection experiment was conducted. Two-day-old chicks were inoculated with JE or WN virus, and challenged with the other virus after 3 weeks. 

**Figure 11:**
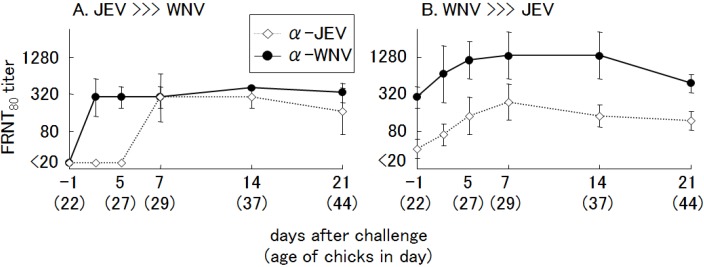
Neutralizing antibody responses in chicks after a secondary challenge with heterologous viruses. Two-day-old chicks (*n =* 4) were inoculated with 100 plaque forming units (PFU) of primary viruses: (**A**) Japanese encephalitis (JE) virus, (**B**) West Nile (WN) virus. After 3 weeks, the chicks (23 days old) were inoculated againwith 1,000 PFU of heterologous virus: (**A**) WN virus, (**B**) JE virus. Anti-JE virus (◇) and -WN virus (●) neutralizing antibody titers were measuredby FRNT_80_ and are expressed as the mean ± SD.

Regardless of which virus was inoculated first, booster immune responses to both homologous and heterologous virus were observed after challenge inoculation (Figure 11). However, it was difficult to judge which virus had infected first, or how many times the chicks were exposed to the viruses, based on the NT.

These results are in agreement with a previous report of combined infections with WN virus and St. Louis encephalitis (SLE) virus [59,60,61], in which the differential diagnosis of those closely related viruses was demonstrated to be very difficult. Fang and Reisen [60] reported that infection with SLE virus after recovery from WN virus infection in house finches elicited a consistent and significant rise in WN virus PRNT titers, but not SLE virus PRNT titers, perhaps because protective immunity prevented the immunologic response associated with a second viremia episode (“original antigenic sin”). This description fits well with our results for infection with JE virus after recovery from WN virus infection. 

Although it is difficult to distinguish the specific flavivirus neutralizing antibodies in multiple infections with heterologous viruses, micro FRNT is able to differentiate antibodies against WN virus and JE virus in single infection. Therefore, micro FRNT is a quite useful method to conduct serological survey in the area where WN and JE viruses are both prevalent.

## 3. Seroprevalence of WN Virus in Wild Birds in Far Eastern Russia

Field surveys were conducted in Far East Russia in 2005 and 2006 to know the seroprevalence of WN virus in wild birds using micro FRNT. Neutralizing antibody to WN virus was identified in 21 serum samples taken from 145 wild birds (14.5%) (Table 2) [62]. Birds that were positive for antibodies to WN virus were in the orders *Anseriformes*, *Charadriiformes*, *Columbiformes*, and *Pelecaniformes*. Birds in these orders are known to support WN virus propagation with high levels of viremia, and to serve as efficient amplifying hosts for the transmission of WN virus to mosquitoes [9]. The JE virus is endemic to East Asia, and is closely related to WN virus. These viruses often show antigenic cross-reactivity in serological tests [30,31]. Therefore, WN virus-positive samples were further tested for the neutralizing antibody to JE virus. The majority of WN virus-positive sera were negative for neutralizing antibody against the JE virus. These data indicate that the positive results of the FRNT for WN virus were caused by antibodies specific to WN virus infection and not because of cross-reactivity with antibodies produced by JE virus infection. 

**Table 2:** Seroprevalence of wild birds collected in Far Eastern Russia (2005–2006) with WNV and/or JEV neutralizing antibodies.

Area/Year	Bird Species (Order)	No. of WNV-Positive/Tested Sera	Positive for Anti-WNV Antibodies %	FRNT_80_ Titer * Range	
				WNV	JEV
Khanka Lake/2005	*Anas poecilorhyncha* (Anseriformes)	1/1	100	160	40
	*Larus ridibundus* (Charadriiformes)	1/1	100	160	80
	*Streptopelia orientalis* (Columbiformes)	1/1	100	1,280	<40
	Five other species	0/23	0	<160	NT ^†^
Anyui River/2005	*Histrionicus histrionicus* (Anseriformes)	3/13	23.1	160–320	40
	Four other species	0/11	0	<160	NT
Khanka Lake/2006	*Anas poecilorhyncha* (Anseriformes)	1/2	50.0	160	40
	*Mergus serrator* (Anseriformes)	1/8	12.5	160	<40
	*Sterna hirundo* (Charadriiformes)	2/13	15.4	160–320	40–320
	*Columba livia* (Columbiformes)	1/1	100	320	80
	*Streptopelia orientalis* (Columbiformes)	4/9	44.4	1,280–2,560	80
	Three other species	0/8	0	<160	NT
Chor River/2006	*Anas poecilorhyncha* (Anseriformes)	2/9	22.2	160	40–80
	*Mergus serrator* (Anseriformes)	2/22	9.1	160–640	40–80
	*Phalacrocorax carbo* (Pelecaniformes)	2/9	22.2	160	40
	Twelve other species	0/14	0	<160	NT
	Total	21/145	14.5	<160–2,560	<40–320

Note: * FRNT80, 80% focus reduction neutralization test; WNV, West Nile virus; JEV, Japanese encephalitis virus; ^†^ NT, Not tested.

All of the rock doves ( *Columba livia* ) tested and some eastern turtle doves (*Streptopelia orientalis*), which are resident birds, had WN virus antibodies and were probably infected with the virus near Khanka Lake. Because Khanka Lake lies far to the east of where WN was first isolated in Russia, the WN virus appears to have been transmitted among wild birds in Far Eastern Russia. The other WN virus-positive birds identified in this study were spotbills (*Anas poecilorhyncha*), harlequin ducks (*Histrionicus histrionicus*), red-breasted mergansers (*Mergus serrator*), black-headed gulls (*Larus ridibundus*), and common terns (*Sterna hirundo*), which are all migratory birds, therefore it is possible that these birds were infected with the WN virus in Far Eastern Russia and carried the virus into other regions of East Asia. In the Asia-Pacific region, migratory water birds typically display north-south flying patterns [63]. Long-distance migratory birds use three flyways, the Central Asian-Indian, East Asian-Australasian, and West Pacific flyways. Among the WN virus antibody-positive bird species, the common tern (*Sterna hirundo*) is a long-distance migratory species that may migrate between Far East Russia and Australasia. The possibility that the WN virus-positive antibodies in common tern might be the result of the Kunjin virus infection could not be excluded because of the limitation of the neutralization test. Recent studies of migration routes of mallard (*Anas platryhynchos*) determined by satellite telemetry have shown that besides the northward flyway from Japan to Russia, a northwestward flyway also exists in Far East Russia [64]. In Japan, WN virus activity has not yet been detected. In the metropolitan area of Tokyo from 2002 to 2006, a total of 7,281 mosquitoes and 139 crow samples (blood, brain, kidney, and spleen) were tested for WN virus RNA, and none of them were positive [65]. In Hokkaido, we also collected about 100 individual wild birds, including crows and sparrows, which were found dead. Kidneys and brains of these birds were tested for WN virus RNA using real-time PCR and none of them were positive [66].

The results of this study suggest that WN virus is distributed throughout Far Eastern Russia and that it may spread to East Asian countries with the migration of wild birds. To prepare for the introduction of WN virus to East Asia, the development of a diagnostic test that can accurately differentiate between WN and JE virus infection is needed. In addition, continued epizootiological evaluation of WN virus infection among birds and humans in Far Eastern Russia and East Asia will be important for monitoring the spread of the disease.

## 4. Conclusions

WN virus causes serious problems in public health since a large numbers of patients with severe encephalitis are reported in various regions of the World. The distribution of the virus is still expanding [67] and the epidemic sometimes upsurges in endemic countries. From 1999 through 2012, a total of 16,196 patients with WN virus neuroinvasive disease were reported and 1,549 patients died in the United States [68]. There is no vaccine for humans and the distribution of WN virus may expand to the area where JE virus is prevalent. Therefore, surveillance using appropriate methods is quite important to know the risk of WN virus infection. Micro FRNT may be a quite useful method to detect WN virus infection in birds and humans in the regions where JE virus is prevalent, especially in eastern Asian countries.

The highly pathogenic WN virus appeared to emerge in the late 1990s and the glycosylation in E protein may be one of the factors of increased pathogenicity of WN virus. The virus replication in birds is enhanced by the glycosylation of E protein and it may result in the increased transmissibility among bird population and higher pathogenicity in birds. It is quite important to know that only one amino acid substitution of the virus may influence the distribution and pathogenicity of WN virus.

Apparently, there is a strong need for vaccine and specific treatments to WN virus infection. To develop these specific measures to WN virus infection, basic research for understanding the replication and pathogenicity of West Nile virus should be further encouraged.
